# Positional Obstructive Sleep Apnea in Children With Down Syndrome: Prevalence, Clinical Characteristics, and Polysomnographic Features

**DOI:** 10.7759/cureus.117723

**Published:** 2026-10-10

**Authors:** Badar Al Dhouyani, Mutasim Abu-Hasan, Haneen M Toma, Mohammed Abushahin, Amal R Al-Naimi

**Affiliations:** 1 Pediatric Pulmonology, Sidra Medicine, Doha, QAT; 2 Pediatrics, University of Lancashire, Preston, GBR

**Keywords:** down syndrome, obstructive sleep apnea, pediatric polysomnography, positional sleep apnea, sleep-disordered breathing

## Abstract

Background: Positional obstructive sleep apnea (POSA), defined as obstructive events that occur predominantly in the supine position, is considered a phenotype of obstructive sleep apnea (OSA) that requires a special diagnostic and therapeutic approach. Children with Down syndrome (DS) are at increased risk of having OSA in general and POSA in particular. However, the clinical and sleep determinants of POSA in DS are not well characterized.

Objective: To determine the prevalence of POSA in children with DS with OSA, compare the clinical and polysomnographic characteristics between POSA and non-POSA phenotypes, and identify predictors of POSA.

Methods: The study is a retrospective evaluation of the entire cohort of children with genetically confirmed DS who completed a diagnostic polysomnography study at the only pediatric sleep lab in Qatar between September 2019 and May 2026. Patients' demographic, clinical, and polysomnographic data were collected from the medical records. Only patients with OSA were included in the analysis. OSA was defined as an obstructive apnea-hypopnea index (AHI) ≥ 1.5 events/hour. OSA was considered positional (POSA) if the ratio of supine to non-supine AHI was ≥2, with at least 20 minutes of sleep recorded in both the supine and non-supine positions. Non-positional (non-POSA) if the ratio was < 2. Comparisons between POSA and non-POSA groups were made using Student's t-test for continuous variables and a chi-square test for categorical variables. Multivariable logistic regression was performed in the study population to identify potential independent predictors of POSA.

Results: A total of 151 children with DS were identified. Only 132 (87%) of the children had OSA. POSA was identified in 61 (46.2%) children and non-POSA in 71 (53.8%) children. There was no significant difference between the two groups in age, gender, weight, height, BMI, or history of snoring or witnessed apnea. There was also no significant difference between the two groups in sleep efficiency, sleep architecture, AHI, OAHI, or OSA severity. Regression analysis showed no significant clinical or PSG predictors of POSA in the study population.

Conclusions: POSA is prevalent in children with DS. However, there are no significant clinical or polysomnographic differences between POSA and non-POSA, suggesting that POSA is not a unique OSA phenotype. Further prospective and interventional studies are needed to confirm the clinical impact of POSA in DS and its treatment.

## Introduction

Obstructive sleep apnea (OSA) is the most common sleep-related breathing disorder in children, affecting approximately 1-4% of the general pediatric population [1]. OSA in children is associated with significant neurocognitive, behavioral, cardiovascular, metabolic, and growth-related consequences, resulting in substantial impairment in health and quality of life [2]. Children with Down syndrome (DS) are particularly susceptible to OSA, with reported prevalence ranging from 30% to 60%, which is substantially higher than those observed in the general pediatric population [3]. This increased prevalence of OSA in DS is due to multiple anatomical and physiological factors common in affected children, including midface hypoplasia, macroglossia, glossoptosis, adenotonsillar hypertrophy, obesity, and generalized hypotonia, which can result in increased susceptibility to upper airway obstruction during sleep [4].

Adenotonsillectomy is the first-line and most effective treatment of OSA in the pediatric population, which is likely to result in complete resolution of symptoms in otherwise healthy children. However, in children with DS, persistent OSA post adenotonsillectomy is common, with more than half of these children reported to have clinically significant disease following surgery [5]. Treatment with continuous positive airway pressure (CPAP) is usually considered for these children with persistent OSA. However, long-term adherence to CPAP can be challenging in children with DS because of the developmental delay, behavioral problems, and caregiver-related factors [6].

Therefore, identifying clinically meaningful OSA phenotypes that help with individualized management that might not necessarily require CPAP therapy has become an important area of pediatric sleep medicine. Positional OSA (POSA) is one of these easily recognizable OSA phenotypes that can be treated simply by adjusting body positioning during sleep. Therefore, identifying POSA in children with DS has potentially important clinical implications.

Body position is an important determinant of upper airway patency during sleep. Gravitational effects in the supine position can cause posterior displacement of the tongue and the surrounding upper airway structures, leading to increased airway collapsibility and increased frequency and severity of obstructive respiratory events. OSA is usually considered POSA if the obstructive respiratory events occur more frequently or more severely in the supine position than in the non-supine position. POSA has been well recognized in the adult population and has been increasingly described in children and is usually identified by an increased ratio of supine to non-supine obstructive events [7].

Wu et al. reported a POSA prevalence of 38.2% in a cohort of children with OSA and demonstrated that POSA prevalence was associated with young age and high OSA severity. The unique characteristics suggest that POSA is a separate phenotype of OSA in children [8]. However, other reports of the prevalence and the associated clinical characteristics in the pediatric population remain highly variable because of differences in population studies and diagnostic criteria used for POSA [9].

Children with DS are expected to have a higher prevalence of POSA than the general pediatric population because of their particular susceptibility to positional upper airway obstruction due to their distinctive airway anatomy and airway hypotonia. Previous studies have demonstrated that body position during sleep and sleep stage influence OSA severity in these children. However, the exact prevalence and the clinical determinants of POSA in this patient population remain incompletely understood [10,11].

Some of the available studies of POSA in children with DS have focused on selected subpopulations, such as children with residual OSA post adenotonsillectomy, potentially limiting the generalizability of the study findings [5]. Very few studies have evaluated the clinical and polysomnographic determinants of POSA in this population.

Our primary objective in this study is to estimate the prevalence of POSA in a cohort of children with DS who were diagnosed with OSA by polysomnography (PSG). We also aim to compare the demographic, clinical, and polysomnographic characteristics between POSA and non-POSA. We also aimed to identify potential predictors of POSA in the entire patient cohort.

## Materials and methods

Methods

Study Population

This retrospective study included all children younger than 18 years with genetically confirmed DS who underwent at least one diagnostic overnight polysomnography (PSG) at the pediatric sleep laboratory at Sidra Medicine, Doha, Qatar, between September 2019 and May 2026. Only children with OSA based on PSG findings were included in the analysis. OSA was defined as an obstructive apnea-hypopnea index (OAHI) ≥1.5 events/hour. Patients were also required to have sufficient polysomnographic data to determine body-position-dependent respiratory indices.

Patients with incomplete polysomnographic data, a history of tracheostomy, or previous positive airway pressure therapy (CPAP or bilevel positive airway pressure (BiPAP)) were excluded. When multiple diagnostic studies were available for the same patient, only the first diagnostic study was included.

Data Collection

Demographic, anthropometric, and clinical data were extracted from patients' electronic medical records, including age, sex, height, weight, body mass index (BMI), and BMI percentile. BMI percentiles were calculated using World Health Organization (WHO) age- and sex-specific pediatric growth references. Clinical data included a history of congenital heart disease, pulmonary hypertension, gastroesophageal reflux disease, asthma, allergic rhinitis, and aspiration. Sleep-related clinical data included a history of snoring, witnessed apneas, adenotonsillar hypertrophy, adenotonsillectomy, and persistent snoring following adenotonsillectomy.

Polysomnography

All eligible participants underwent overnight diagnostic PSG using a standardized pediatric protocol. Recorded physiological signals included electroencephalography, electrooculography, chin and bilateral anterior tibialis electromyography, electrocardiography, nasal pressure, oronasal thermal airflow, thoracoabdominal respiratory inductance plethysmography, pulse oximetry, body position, and transcutaneous carbon dioxide (TcCO₂) monitoring.

Sleep staging and respiratory event scoring were manually performed by experienced registered polysomnographic technologists according to the pediatric scoring rules of the American Academy of Sleep Medicine (AASM) Manual for the Scoring of Sleep and Associated Events and were subsequently reviewed and interpreted by pediatric sleep physicians [12].

OSA was defined as an OAHI ≥1.5 events/hour. OSA severity was classified as mild when the OAHI was ≥1.5 but <5 events/hour, moderate when the OAHI was ≥5 but <10 events/hour, and severe when the OAHI was ≥10 events/hour [13].

Sleep efficiency was calculated as total sleep time (TST) divided by total recording time, multiplied by 100. The percentage of sleep time spent in the supine position was calculated as the TST spent in the supine position divided by TST, multiplied by 100. The percentage of TST with peripheral oxygen saturation (SpO₂) <90% was calculated as the cumulative sleep time with SpO₂ <90% divided by TST, multiplied by 100.

Positional OSA Classification

For the positional analysis, participants were required to have at least 20 minutes of sleep in both the supine and non-supine positions. Supine OAHI was calculated as the number of obstructive respiratory events occurring during supine sleep divided by the total supine sleep time in hours. Non-supine OAHI was calculated as the number of obstructive respiratory events occurring during non-supine sleep divided by the total non-supine sleep time in hours.

Children with OSA were classified as having POSA if the ratio of the supine OAHI to the non-supine OAHI was ≥2. Children who did not meet this criterion were classified as having non-POSA.

The prevalence of POSA was calculated as the number of children meeting the POSA criteria divided by the total number of children included in the positional analysis. Demographic, anthropometric, clinical, and polysomnographic characteristics were compared between the POSA and non-POSA groups. The cohort was also evaluated to identify potential predictors of POSA and potential predictors of the supine-to-non-supine OAHI ratio.

Ethical Approval

The study was approved by the Sidra Medicine Institutional Review Board (IRB approval number 2208179-3). The requirement for informed consent was waived because of the retrospective study design and use of de-identified clinical data.

Statistical Analysis

Continuous variables were assessed for normality and are presented as mean ± standard deviation (SD) or median (interquartile range (IQR)), as appropriate. Categorical variables are presented as frequencies and percentages. Comparisons between the POSA and non-POSA groups were performed using the independent-samples t-test for normally distributed continuous variables and the Mann-Whitney U test for non-normally distributed continuous variables. Categorical variables were compared using the chi-square test or Fisher's exact test, as appropriate.

Potential demographic, anthropometric, clinical, and polysomnographic predictors of POSA were initially evaluated using univariable binary logistic regression. Variables considered clinically relevant or associated with POSA in the univariable analyses were subsequently entered into a multivariable binary logistic regression model. Results are reported as odds ratios (ORs) with corresponding 95% confidence intervals (CIs).

Potential predictors of the supine-to-non-supine OAHI ratio were initially evaluated using univariable linear regression. Variables considered clinically relevant or associated with the supine-to-non-supine OAHI ratio in the univariable analyses were subsequently entered into a multiple linear regression model. Regression coefficients (β) with corresponding 95% CIs are reported.

All statistical tests were two-sided, and a P-value < 0.05 was considered statistically significant. Statistical analyses were performed using Statistical Product and Service Solutions (SPSS, version 27.0; IBM SPSS Statistics for Windows, Armonk, NY).

## Results

We identified a total of 151 children with genetically confirmed DS who have completed PSG during the study periods. A total of 132 (87%) of these children had OSA and were included in the analysis. OSA was considered POSA in 61 children (46.2%) and non-POSA in 71 (53.8%) children.

Demographic, anthropometric characteristics were similar between the two groups. There was no significant difference in age, gender, weight, height, body mass index (BMI), or BMI percentile. There was also no significant difference in history of habitual snoring, witnessed apnea, adenotonsillar hypertrophy, previous adenotonsillectomy, or persistent OSA following adenotonsillectomy. There was no difference in frequency of associated comorbidities, including congenital heart disease, gastroesophageal reflux disease, aspiration, asthma, allergic rhinitis, and pulmonary hypertension (Table 1).

**Table 1:** Demographic and Clinical Characteristics of Children With POSA and Non-POSA. Continuous variables are presented as mean ± standard deviation (SD), and categorical variables are presented as number (percentage). Normally distributed continuous variables were compared using the independent-samples t-test. Categorical variables were compared using Pearson's chi-square test when expected cell counts were adequate and Fisher's exact test when expected cell counts were <5. For Fisher's exact test, a test statistic is not applicable (N/A). The statistical test applied to each variable is specified in the table. All tests were two-sided, and a p-value <0.05 was considered statistically significant. Abbreviations: BMI, body mass index; N/A, not applicable; OSA, obstructive sleep apnea; POSA, positional obstructive sleep apnea; SD, standard deviation

Variable	POSA (N=61)	Non-POSA (N=71)	Test statistic	P-value	Statistical test
Age (years), mean ± SD	6.5 ± 3.9	5.9 ± 3.6	t = 0.85	0.39	Independent-samples t-test
Female, n (%)	20 (33%)	28 (39%)	χ² = 0.63	0.43	Pearson’s chi-square test
BMI (kg/m²), mean ± SD	20.5 ± 5.9	19.6 ± 5.8	t = 0.78	0.44	Independent-samples t-test
BMI percentile, mean ± SD	80.7 ± 24.0	72.7 ± 35.2	t = 1.13	0.26	Independent-samples t-test
History of snoring, n (%)	49 (80%)	57 (80%)	χ² <0.01	>0.99	Pearson’s chi-square test
History of witnessed sleep apnea, n (%)	23 (38%)	30 (42%)	χ² = 0.21	0.65	Pearson’s chi-square test
Congenital heart disease, n (%)	35 (57%)	37 (52%)	χ² = 0.37	0.55	Pearson’s chi-square test
Gastroesophageal reflux disease, n (%)	6 (10%)	13 (18%)	χ² = 1.90	0.17	Pearson’s chi-square test
Aspiration, n (%)	14 (23%)	25 (35%)	χ² = 2.40	0.12	Pearson’s chi-square test
Asthma, n (%)	4 (7%)	8 (11%)	χ² = 0.88	0.35	Pearson’s chi-square test
Allergic rhinitis, n (%)	1 (2%)	6 (8%)	N/A	0.12	Fisher’s exact test
Pulmonary hypertension, n (%)	1 (2%)	7 (10%)	N/A	0.07	Fisher’s exact test
Adenotonsillar hypertrophy, n (%)	30 (49%)	49 (69%)	χ² = 2.22	0.14	Pearson’s chi-square test
History of adenotonsillectomy, n (%)	15 (25%)	16 (23%)	χ² = 0.15	0.70	Pearson’s chi-square test

Polysomnographic characteristics were also similar between both groups. There was no significant difference in sleep efficiency, REM percentage, AHI, OAHI, RE- OAHI, oxygen desaturation index (ODI), mean oxygen saturation, oxygen saturation nadir, and the percentage of total sleep time with oxygen saturation below 90% (Table 2).

**Table 2:** Comparison of Polysomnographic Characteristics Between Children With Positional and Non-Positional Obstructive Sleep Apnea (N = 132). Legend: Continuous variables are presented as mean ± standard deviation (SD), and categorical variables as number (percentage). Continuous variables were compared using the independent-samples t-test, and categorical variables were compared using Pearson’s chi-square test. OSA severity was analyzed as a single three-category variable (mild, moderate, and severe); therefore, χ² = 1.83 and p = 0.40 represent the overall comparison of OSA severity distribution between the POSA and non-POSA groups and do not represent a separate comparison of the mild OSA category. Separate statistical tests were not performed for each individual OSA severity category. All tests were two-sided, and a P-value <0.05 was considered statistically significant. Abbreviations: AHI, apnea-hypopnea index; N/A, not applicable; OAHI, obstructive apnea-hypopnea index; ODI, oxygen desaturation index; OSA, obstructive sleep apnea; POSA, positional obstructive sleep apnea; PSG, polysomnography; REM, rapid eye movement; SD, standard deviation; SpO₂, peripheral oxygen saturation; TST, total sleep time * The χ² statistic and P-value shown on the mild OSA row represent the overall three-category comparison of OSA severity, not a separate comparison of mild OSA.

Variable	POSA (n=61)	Non-POSA (n=71)	Test statistic	P-value	Statistical test
Sleep efficiency (%), mean ± SD	78.0 ± 15.5	78.9 ± 12.0	t = −0.34	0.73	Independent-samples t-test
Time in supine position (%), mean ± SD	49.5 ± 24.4	42.1 ± 27.6	t = 1.62	0.11	Independent-samples t-test
REM sleep (%), mean ± SD	22.3 ± 7.7	23.9 ± 7.5	t = −1.20	0.23	Independent-samples t-test
AHI (events/hour), mean ± SD	10.8 ± 9.7	9.8 ± 10.2	t = 0.56	0.58	Independent-samples t-test
OAHI (events/hour), mean ± SD	9.8 ± 9.2	9.2 ± 10.2	t = 0.35	0.73	Independent-samples t-test
REM OAHI (events/hour), mean ± SD	22.2 ± 21.1	17.4 ± 17.5	t = 1.43	0.16	Independent-samples t-test
Mean SpO₂ (%), mean ± SD	96.0 ± 2.0	96.3 ± 1.7	t = −0.73	0.46	Independent-samples t-test
SpO₂ nadir (%), mean ± SD	85.6 ± 6.3	85.9 ± 6.1	t = −0.29	0.77	Independent-samples t-test
TST with SpO₂ <90% (%), mean ± SD	1.3 ± 3.1	1.2 ± 4.0	t = 0.06	0.95	Independent-samples t-test
ODI (events/hour), mean ± SD	15.0 ± 13.0	15.5 ± 12.3	t = −0.23	0.82	Independent-samples t-test
Snoring during PSG, n (%)	40 (66%)	49 (69%)	χ² = 0.05	0.81	Pearson’s chi-square test
OSA severity, n (%)					
Mild	26 (43%)	29 (41%)	χ² = 1.83	0.40	Pearson’s chi-square test*
Moderate	13 (21%)	22 (31%)	N/A	N/A	N/A
Severe	22 (36%)	20 (28%)	N/A	N/A	N/A

Children with POSA spent a slightly longer proportion of sleep time in the supine position, but it was not statistically significant (49.5% vs. 42.7%, p = 0.08). In addition, children with POSA had slightly higher REM OAHI values than non-POSA, but this was not statistically significant (22.1 ± 21.0 vs. 17.4 ± 17.0 events/hour, p = 0.17). OSA severity was also similar between the two groups (p = 0.43).

Logistic regression analysis showed no significant independent predictors of POSA (Table 3). In addition, there was no significant predictor of a high supine/non-supine OAHI ratio (Table 4).

**Table 3:** Multivariable Binary Logistic Regression Analysis of Potential Predictors of Positional Obstructive Sleep Apnea in Children With Down Syndrome and Obstructive Sleep Apnea. A multivariable binary logistic regression analysis was performed with positional obstructive sleep apnea (POSA) as the binary dependent variable (POSA = 1; non-POSA = 0). All predictors listed in the table were entered simultaneously into the model. The β-coefficient represents the change in the log-odds of POSA associated with a one-unit increase in a continuous predictor or, for Gender, female compared with male as the reference category. Odds ratios (ORs) are presented with corresponding 95% confidence intervals (CIs). Age was modeled per one-year increase; BMI per 1 kg/m² increase; sleep efficiency, REM sleep, and supine sleep time per one-percentage-point increase; and AHI, OAHI, and REM OAHI per one event/hour increase. The analysis was performed using complete cases and included 128 children, with 58 POSA events; four participants were excluded because of missing predictor data. All tests were two-sided, and a p-value <0.05 was considered statistically significant. Abbreviations: AHI, apnea-hypopnea index; BMI, body mass index; CI, confidence interval; OAHI, obstructive apnea-hypopnea index; OR, odds ratio; OSA, obstructive sleep apnea; POSA, positional obstructive sleep apnea; REM, rapid eye movement; SE, standard error

Predictor	Unit/Reference category	β	SE	OR	95% CI for OR	P-value
Age	Per 1-year increase	0.038	0.062	1.04	0.92-1.17	0.54
Gender	Female vs male	−0.230	0.393	0.79	0.37-1.72	0.56
BMI	Per 1 kg/m² increase	0.010	0.039	1.01	0.93-1.09	0.80
Sleep efficiency	Per 1-percentage-point increase	0.009	0.016	1.01	0.98-1.04	0.56
REM sleep	Per 1-percentage-point increase	−0.029	0.029	0.97	0.92-1.03	0.32
AHI	Per 1 event/hour increase	0.235	0.153	1.27	0.94-1.71	0.12
OAHI	Per 1 event/hour increase	−0.275	0.162	0.76	0.55-1.04	0.09
REM OAHI	Per 1 event/hour increase	0.023	0.017	1.02	0.99-1.06	0.16
Supine sleep time	Per 1-percentage-point increase	0.001	0.008	1.00	0.99-1.02	0.94

**Table 4:** Multiple Linear Regression Analysis of Potential Predictors of the Supine-to-non-Supine Obstructive Apnea-Hypopnea Index Ratio in Children With Down Syndrome and Obstructive Sleep Apnea. Multiple linear regression was performed with the supine-to-non-supine OAHI ratio as the dependent variable. All predictors shown in the table were entered simultaneously into the model. β represents the unstandardized regression coefficient, corresponding to the expected change in the supine-to-non-supine OAHI ratio for a one-unit increase in a continuous predictor or for females compared with males as the reference category, while holding all other variables constant. Age was modeled per one-year increase; BMI per 1 kg/m² increase; sleep efficiency, REM sleep, and supine sleep time per 1-percentage-point increase; and AHI, OAHI, and REM OAHI per 1 event/hour increase. The complete-case analysis included 128 children. Overall model statistics were R² = 0.087, adjusted R² = 0.017, F(9,118) = 1.251, p = 0.271. All tests were two-sided, and a p-value <0.05 was considered statistically significant. Abbreviations: AHI, apnea-hypopnea index; BMI, body mass index; CI, confidence interval; OAHI, obstructive apnea-hypopnea index; OSA, obstructive sleep apnea; REM, rapid eye movement; SE, standard error

Predictor	Unit/Reference category	β	SE	95% CI for β	P-value
Age	Per 1-year increase	0.430	0.222	−0.011 to 0.870	0.056
Geneder	Female vs male (reference)	−1.990	1.410	−4.782 to 0.802	0.161
BMI	Per 1 kg/m² increase	−0.040	0.140	−0.317 to 0.237	0.776
Sleep efficiency	Per 1-percentage-point increase	0.070	0.060	−0.049 to 0.189	0.245
REM sleep	Per 1-percentage-point increase	−0.080	0.100	−0.278 to 0.118	0.425
AHI	Per 1 event/hour increase	0.530	0.460	−0.381 to 1.441	0.251
OAHI	Per 1 event/hour increase	−0.690	0.490	−1.660 to 0.280	0.162
REM OAHI	Per 1 event/hour increase	0.100	0.060	−0.019 to 0.219	0.098
Supine sleep time	Per 1-percentage-point increase	−0.030	0.030	−0.089 to 0.029	0.319

## Discussion

In this retrospective cohort study of children with DS who were diagnosed with OSA by polysomnography, nearly half (46.2%) had POSA. Despite the high prevalence of POSA, demographic characteristics, clinical features, sleep characteristics, and OSA severity were not significantly different between children with POSA and children with non-POSA. Furthermore, regression showed no independent predictors of POSA or a high supine-to-non-supine OAHI ratio. These findings suggest that, although POSA is common among children with DS, it does not seem to be distinguished based on demographic, anthropometric, or clinical features. It also cannot be distinguished based on clinical or sleep characteristics or even OSA severity. Taken together, these findings suggest that, within our cohort, POSA was not associated with a readily identifiable demographic, anthropometric, clinical, or polysomnographic phenotype among children with DS and OSA.

Previous studies in the pediatric population have demonstrated that supine position can increase the frequency and severity of obstructive respiratory events. In the supine position, gravitational effects can result in posterior displacement of the tongue and the surrounding soft tissues, thereby increasing upper airway collapsibility. However, the reported prevalence and characteristics of pediatric POSA vary considerably depending on the population studied and the diagnostic criteria applied. Wang et al. reported that POSA represents a common phenotype among children with OSA and identified several clinical characteristics associated with positional dependency [10]. Walter et al. similarly demonstrated that body position influences obstructive respiratory events in children with OSA [9]. In adults, Menon et al. systematically reviewed the relationship between body position and OSA and highlighted considerable heterogeneity in the definitions, populations, and methodologies used across positional OSA studies [7].

In children with DS, body position can interact with multiple anatomical and physiological mechanisms that influence upper airway narrowing and collapsibility during sleep. Senthilvel et al. described characteristic forward-leaning sleep postures in children with DS and suggested that these unusual positions may represent a compensatory mechanism to improve upper airway patency during sleep [12]. Subsequently, Nisbet et al. demonstrated that both body position and sleep state influence OSA severity in children with DS, highlighting the complex interaction between sleep physiology and upper airway obstruction [14]. Santoro et al. also reported distinctive sleep-position patterns in children with DS and OSA, including a predominance of lateral/decubitus sleep and relatively little time spent supine, suggesting that preferential non-supine positioning may represent an adaptive response to upper airway obstruction [15]. Collectively, these findings support an important interaction between sleep position and the expression of OSA in children with DS.

More recently, Don et al. reported that children with DS have greater OSA severity across different body positions and sleep stages than children without DS but are less likely to exhibit the classic positional OSA phenotype [16]. These findings differ from the studies suggesting positional OSA in DS. These differences in study conclusions reflect differences in the populations studied, OSA severity, previous treatments, and the definition used for POSA. For example, Xiao et al. specifically evaluated children with DS who had residual OSA following adenotonsillectomy and found that POSA was common in this selected subpopulation [17]. These differences emphasize the importance of considering the clinical characteristics of the population when interpreting the prevalence and significance of POSA.

Our study differs from previous reports by evaluating a cohort of all children with DS who have OSA, at any degree of severity, rather than restricting the analysis to children with residual OSA following adenotonsillectomy only. Our findings, therefore, provide complementary insight into the prevalence and characteristics of POSA across the broad clinical spectrum of children with DS and OSA.

We found no significant differences between POSA and non-POSA groups with respect to age, sex, BMI, BMI percentile, clinical symptoms, comorbidities, sleep efficiency, sleep architecture, or overall OSA severity. Furthermore, no independent predictors of POSA were identified after adjustment for potential confounders. Similarly, no significant predictors of the supine-to-non-supine OAHI ratio were identified. These findings suggest that positional dependency in children with DS is unlikely to be explained by a single readily identifiable anatomical, physiological, or clinical factor and may instead reflect the combined effects of multiple mechanisms contributing to upper airway collapsibility.

Despite the absence of identifiable clinical or polysomnographic predictors of POSA, recognizing positional dependency may still have potential clinical relevance. Identification of POSA could help inform individualized management strategies, particularly in children with residual OSA following adenotonsillectomy or in those who have difficulty tolerating positive airway pressure therapy.

Recently, a randomized controlled crossover trial of positional therapy in children with POSA demonstrated a significant reduction in supine sleep time, although the reduction in OAHI did not reach statistical significance across the entire cohort, suggesting heterogeneity in treatment response [17]. These findings support further investigation of the effectiveness of positional therapy in selected pediatric patients, which remains limited. Prospective studies are needed to determine whether targeted positional interventions can provide clinically meaningful and sustained improvements in OSA severity in this patient population.

This study has several limitations that should be considered when interpreting the findings. First, its retrospective and single-center design may limit the generalizability of the results to other populations of children with DS. Second, the relatively limited sample size, particularly after stratification into POSA and non-POSA groups, may have reduced the statistical power to detect small differences between groups or identify independent predictors of POSA. In addition, positional classification was based on a single-night polysomnographic study and may not fully reflect habitual sleep position or night-to-night variability. Therefore, the absence of statistically significant predictors should not be interpreted as definitive evidence that demographic, clinical, or polysomnographic factors have no association with POSA. Larger, prospective, multicenter studies are needed to validate these findings and further characterize the clinical significance of POSA in children with DS.

## Conclusions

In this retrospective cohort, POSA was highly prevalent among children with DS and OSA. However, no demographic, clinical, or polysomnographic variables were identified that reliably distinguished children with POSA from those without POSA. These findings suggest that, within our cohort, positional OSA was not associated with a clinically distinctive phenotype or necessarily with more severe disease. Recognition of positional OSA may still facilitate individualized management strategies and identify patients who could benefit from future positional interventions. Given the retrospective, single-center design and relatively limited sample size, prospective multicenter studies are needed to validate these findings and determine the effectiveness of positional therapy in this high-risk population.
